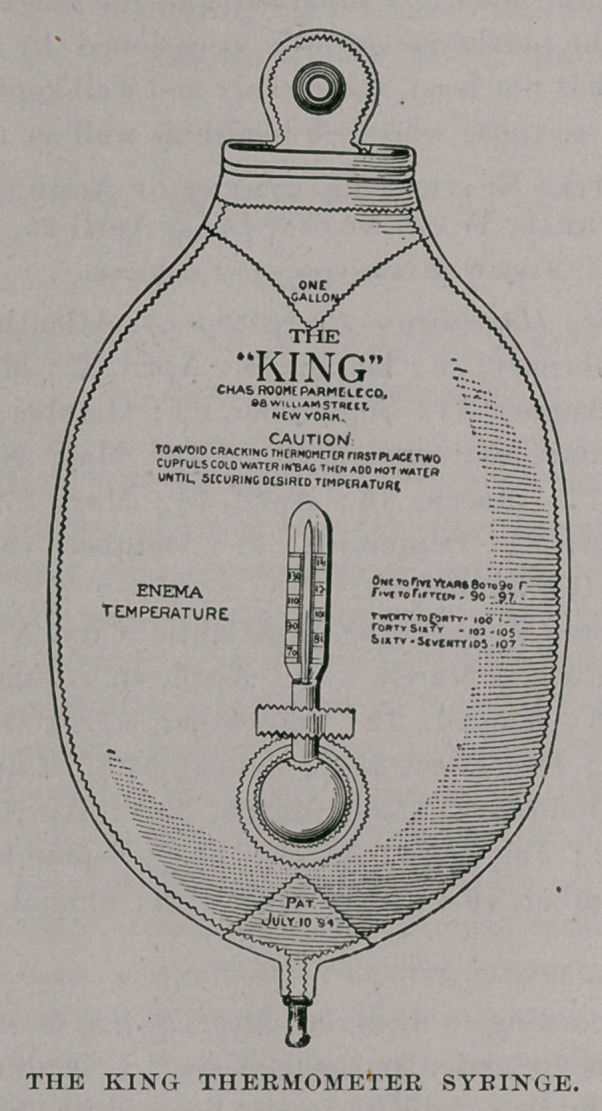# The King Thermometer Syringe

**Published:** 1895-07

**Authors:** 


### New Instrument.

The importance of properly regulating the temperature of enemas and douches is well understood by most clinicians. This applies to children as well as adults. The inconvenience of regulating temperature by ordinary methods has led to the construction of the King gravity syringe, which is here illustrated. With this appara-

THE KING THERMOMETER SYRINGE.

tus, equipped with a double current soft rubber rectal tube, the colon can be flushed with ease, provided the patient is properly postured. It is made of the best quality of rubber, holds one gallon of water, and with the thermometer attachment costs only $2.25. Physicians will readily appreciate the value of this device known as the King thermometer syringe. The Charles Roome Parmele Co., 98 William street, New York, will supply this syringe through instrument dealers and druggists.

## Figures and Tables

**Figure f1:**